# Clinical utility of whole-genome sequencing in identifying a rare *Mycobacterium* species causing native joint infection

**DOI:** 10.1128/asmcr.00062-26

**Published:** 2026-09-23

**Authors:** M. Globan, J. Moselen, N. Wang, E. Steinig, Y. Nong, L. Viberg, L. Caly, K. A. Bond

**Affiliations:** 1 Victorian Infectious Diseases Reference Laboratory, Royal Melbourne Hospital, at the Peter Doherty Institute for Infection and Immunity 534133, Melbourne, Victoria, Australia; 2 Department of Microbiology and Immunology, University of Melbourne, Melbourne, Victoria, Australia; 3 Department of Infectious Diseases, Royal Children’s Hospital, Melbourne, Victoria, Australia; 4 Department of Infectious Diseases, University of Melbourne, Melbourne, Victoria, Australia; 5 Department of Biochemistry and Molecular Biology, Monash University 198101, Clayton, Victoria, Australia; 6 Department of Microbiology, Royal Melbourne Hospital 90134, Melbourne, Victoria, Australia; 7 Department of Infectious Diseases, Royal Melbourne Hospital 90134, Melbourne, Victoria, Australia; Rush University Medical Center, Chicago, Illinois, USA

**Keywords:** non-tuberculous mycobacteria (NTM), bone and joint, diagnostics, whole-genome sequencing, Illumina, Oxford Nanopore Technologies, pediatric

## Abstract

**Background:**

The gold standard for identifying non-tuberculous mycobacteria (NTM) is partial sequencing of one or more distinct gene loci and determining the closest match through reference database matching. The advent of whole-genome sequencing (WGS) allows for higher accuracy in resolving *Mycobacterium* species, by matching a larger genome, although at increased cost. Determining when to employ WGS for NTM identification in diagnostic laboratories is a challenge, particularly when only a minority of isolated NTM are clinically significant.

**Case Summary:**

A 5-year-old patient with inflammatory arthritis, previously responsive to intra-articular glucocorticoid injections, presented with a chronic, painful, hot, swollen left knee. An NTM isolated from synovial fluid was initially reported as *Mycobacterium intracellulare* subsp. *chimaera*, based on gene-loci 16S rRNA and internal transcribed spacer (rRNA 16S-23S ITS) PCR and sequencing. WGS subsequently determined the NTM to best match a single, novel *Mycobacterium* species collected from a patient with lung disease from South Korea, 2018 (NCBI upload 2022). Phylogenetic analysis determined the isolate from our case to share the most recent ancestry with *Mycobacterium nebraskense*, and clustered in a sister clade to that of *Mycobacterium avium intracellulare* complex. The patient’s symptoms improved after a prolonged course of rifabutin, azithromycin, and moxifloxacin, with samples culture-negative 7 months following treatment.

**Conclusion:**

We describe a rare NTM as the cause of a significant joint infection in a 5-year-old child. WGS was instrumental in defining the diagnosis and should be considered early for accurate NTM identification in significant infections, those non-responsive to treatment, or where iatrogenic or outbreak-related cases are a consideration.

## INTRODUCTION

Accurate species-level identification of non-tuberculous mycobacteria (NTM) is crucial for clinical management, as species attribution often guides therapy. *In vitro* susceptibility testing remains vital for rapidly growing mycobacteria. In other species where treatment regimens are uniform, such as the *Mycobacterium avium* complex (MAC), precise identification distinguishes reinfection from reactivation (1–3). Mycobacterial species-level identification relies on genomic loci, such as rRNA (16S rRNA and/or internal transcribed spacer [ITS]), and *rpoB* and *hsp65* genes (2). However, single-locus sequencing fails to resolve 20%–50% of the ~200 NTM species, limiting diagnostic accuracy (4, 5). While multi-locus sequence analysis improves resolution, it increases complexity, cost, and turnaround time.

Whole-genome sequencing (WGS) has become the global standard for *Mycobacterium tuberculosis*, enabling precise strain typing and resistance prediction (6–8). In contrast, NTM WGS pipelines and reference databases remain underdeveloped, with limited bioinformatic tools and sparse representation of NTM species in databases such as NCBI RefSeq and GenBank. Here, we describe a case where WGS confirmed an uncommon mycobacteria species in a joint infection, improving speciation accuracy and clinical clarity over standard gene locus assays.

## CASE PRESENTATION

In October 2021, a 5-year-old Australian-born female was diagnosed with anti-nuclear antibody-positive oligoarticular juvenile idiopathic arthritis, involving her knees and ankles. She was previously healthy and fully immunized with no significant medical history. Following diagnosis, her inflammatory arthritis was successfully controlled with regular intra-articular corticosteroid injections. In October 2022, after her sixth injection, her left knee remained significantly inflamed with active synovitis, despite clinical remission elsewhere. This prompted a trial of systemic immunosuppression with methotrexate (March–July 2023), without significant improvement.

**Fig 1:**
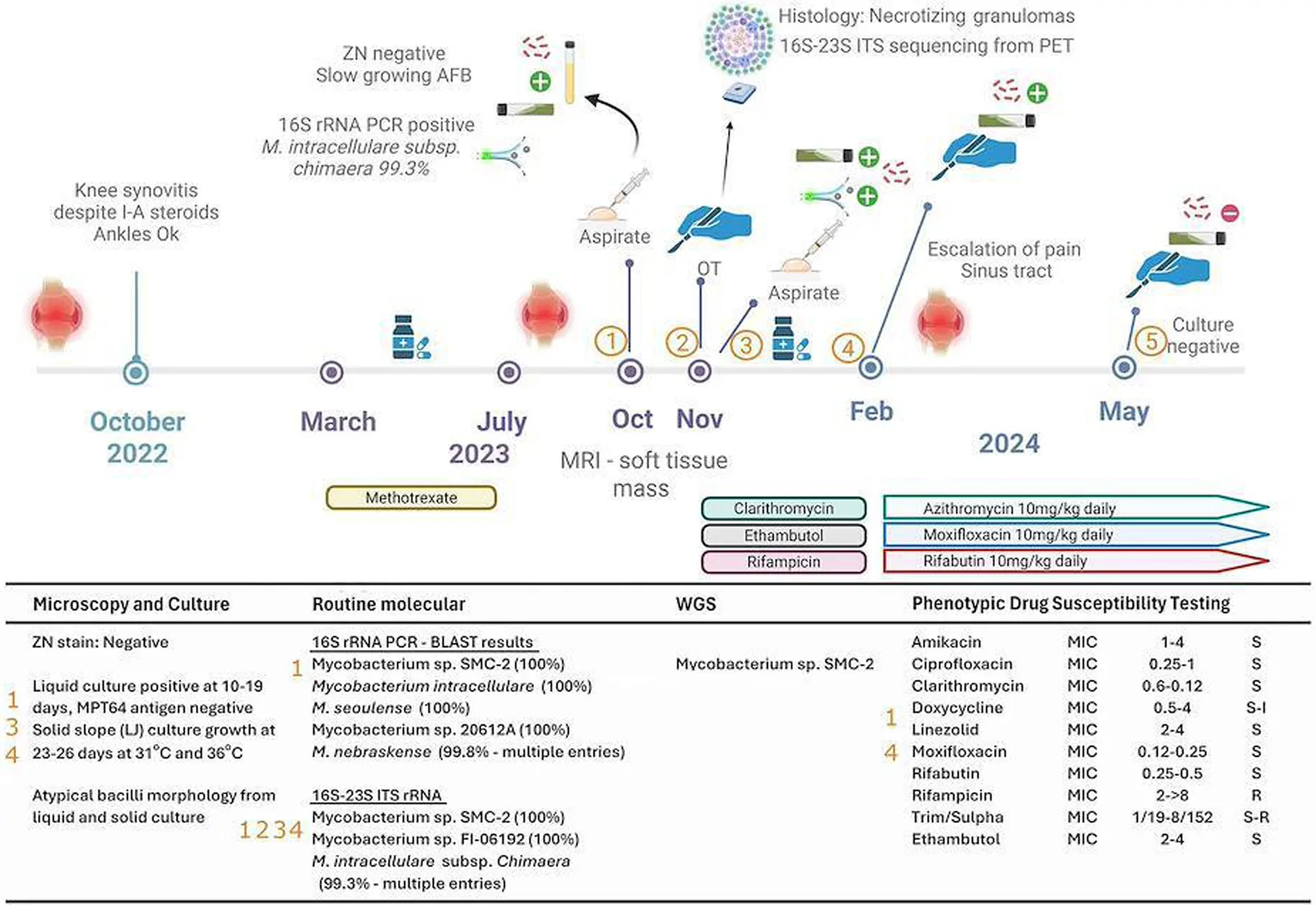
Timeline of events and corresponding microbiological results. Numbers 1–5 on timeline refer to sampling time points; corresponding numbers in the microbiological table correspond to results from those time points. Green circles with + sign indicate positive culture; red circles with – sign indicate negative cultures. I-A, intra-articular steroids; ZN, Ziehl-Neelsen; AFB, acid-fast bacilli; OT, operation, PET, paraffin-embedded tissue. ^#^ Multiple replicates performed, MIC values are given in µg/mL; CLSI breakpoints for slow-growing mycobacteria applied; Trim/Sulpha, trimethoprim/sulphamethoxazole; S, susceptible; I, intermediate; R, resistant. Created in BioRender. Bond, K. (2025) https://BioRender.com/ccse6in.

In response to escalating symptoms, a left knee MRI in October 2023 revealed a soft tissue mass, leading to surgical excision (Fig. 1). Histology showed necrotizing granulomatous inflammation, with acid-fast bacilli (AFB) detected on Ziehl-Neelsen (ZN) and Fite stains. Synovial fluid with few AFB seen on microscopy was culture-positive for mycobacteria. Pan-mycobacterial PCR (target rRNA 16S-23S ITS) and sequencing performed directly on the fluid initially reported *Mycobacterium intracellulare* subsp. *chimaera*. In the context of these results, the patient underwent a knee washout and residual tissue excision in November 2023, with repeat culture confirming the NTM.

The patient commenced on clarithromycin (12.5 mg/kg BD), ethambutol (15 mg/kg daily), and rifampicin (15 mg/kg daily). After 3 months, symptoms worsened with escalation of knee pain, erythema and warmth, fluid re-accumulation, and evolution of a sinus tract evident on MRI. Histology of fresh tissue from a third procedure (February 2024) confirmed necrotizing granulomatous inflammation, with a positive pan-mycobacterial PCR (target rRNA 16S-23S ITS) and sequencing results consistent with the knee fluid. Mycobacterial culture remained positive. Antimicrobial therapy was changed to rifabutin (10 mg/kg daily), azithromycin (10 mg/kg daily), and moxifloxacin (10 mg/kg daily), based on susceptibility results, previous experience with using similar regimens for NTM, and pediatric tolerability. Though the medication was well tolerated overall, the patient eventually developed generalized abdominal discomfort and a reduction in appetite (without vomiting, diarrhea or other gastrointestinal sequelae). Treatment was discontinued after 10 months, with excellent adherence throughout. Repeat sampling 7 months post-diagnosis was culture-negative.

Formal immunological workup revealed no underlying immunodeficiency. Given the localized disease at a site associated with repeated corticosteroid injections, *Mycobacterium* infection was thought to most likely be iatrogenic in origin, with no other cases identified among patients receiving similar injections.

### Microbiological methods

Routine phenotypic mycobacterial processing followed CLSI guidelines, including ZN staining, culturing untreated and decontaminated samples onto solid (Lowenstein-Jensen with pyruvate, Brown and Buckle, 12 weeks, 31°C and 36°C) and liquid media (BD Bactec MGIT system) (Fig. 1) (2). Primary sample DNA extraction used glass bead homogenization and QIAamp DNA Minikit (Qiagen) as per manufacturer’s instructions. Cultured isolates underwent mechanical lysis (FastPrep-24, MP Biomedicals), followed by purification with the QIAamp 96 DNA QIAcube HT kit (Qiagen) as per manufacturer’s instructions.

Pan-mycobacterial PCR was performed on knee fluid, fresh and fixed tissue targeting the rRNA 16S-23S ITS region (9), followed by Sanger sequencing and a BLASTn search of the 220-bp amplicon against the GenBank database (10). GenBank results were ambiguous, returning hits to *Mycobacterium* sp. SMC-2, *Mycobacterium* sp. FI-06192, and *M. intracellulare* subsp. *chimaera*. An initial identification of *M. intracellulare* subsp. *chimaera* was made based on match frequency over single hit matches (Fig. 1). The inability to rule out other species limited diagnostic confidence. Consequently, 16S rRNA PCR, sequencing, and a BLASTn search were performed on an isolate (11) returning hits of *Mycobacterium* sp. SMC-2, *M. intracellulare*, and *Mycobacterium seoulense*. Whole-genome sequencing was subsequently performed to provide a definitive identification.

Phenotypic drug susceptibility testing was performed on isolates from time points 1 and 4, using broth microdilution (Thermo Fisher SLOWMYCO1). Minimum inhibitory concentrations (MICs, μg/mL) were determined by visual reading at 7 and 14 days by two independent scientists.

### Whole-genome sequencing investigation

**Fig 2:**
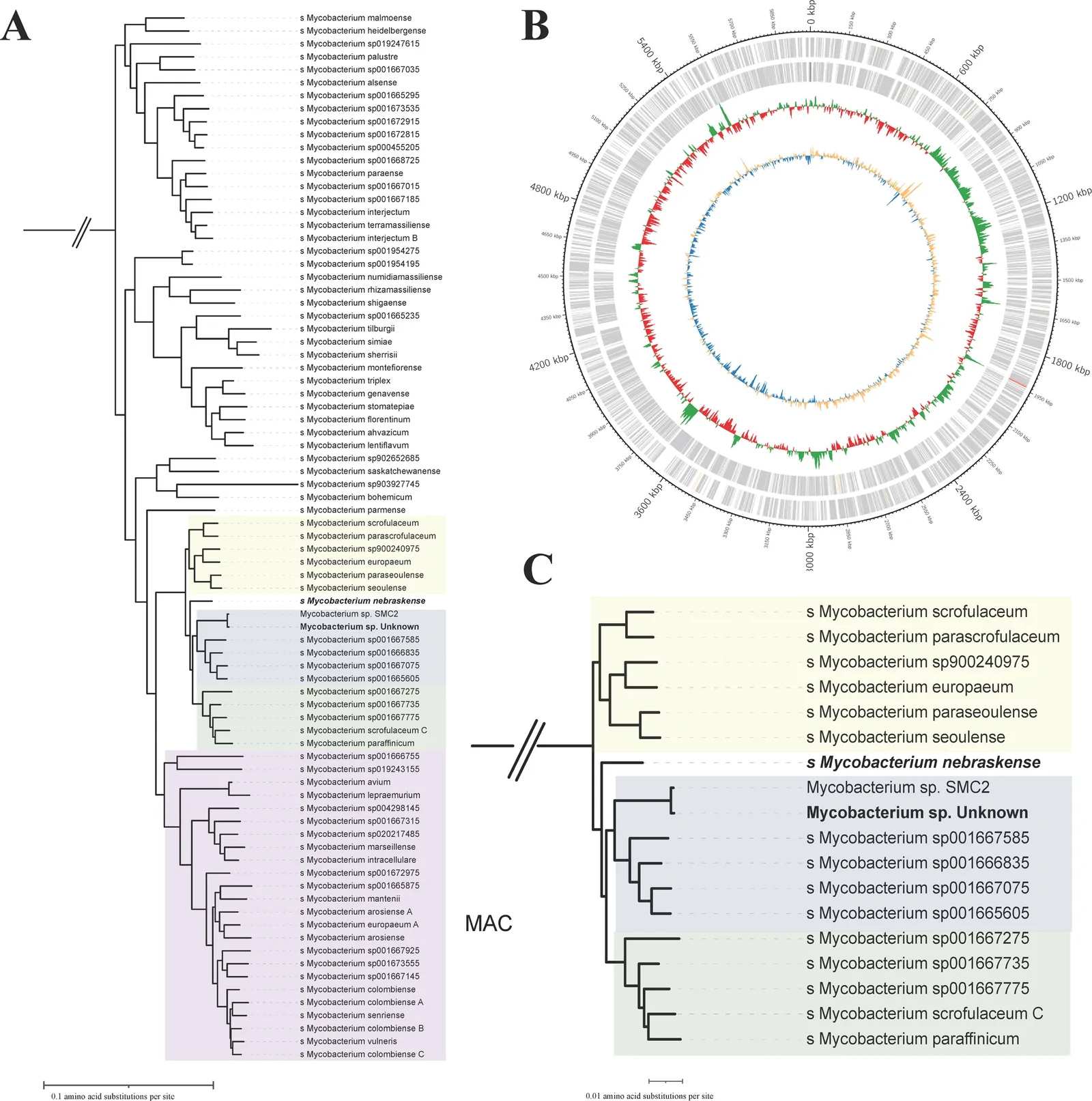
Genomic analysis. (**A**) *De novo* construction of the bacterial reference phylogeny (GTDB) including the completed reference genome of *Mycobacterium* sp. unknown (bold). The phylogeny was subset to the clade consisting of the closest related species within the genus *Mycobacterium* (dashes on the root branch). Purple-shaded area of the tree indicates the clade to which *Mycobacterium avium* subsp. *chimaera* belongs (*Mycobacterium avium* complex, MAC). (**B**) Closed genome assembly from the hybrid assembly of ONT long reads and Illumina short reads. Inner circle represents the GC skew (orange: positive; blue: negative) that highlights the inflection points at the origin of replication and its opposite region, indicating a circular and complete assembly of the chromosome. Outer circle represents deviations of GC content above (green) and below (red) the chromosomal average. Gray segments in the outer track indicate predicted coding regions on the positive and negative strand. (**C**) Clade structure of the closest relatives of *Mycobacterium* sp. unknown extracted from the phylogeny in panel A. Yellow and green boxes highlight the monophyletic sister clades to the clade containing *Mycobacterium* sp. SMC-2 (blue) as well as its most recent common ancestor *M. nebraskense* (bold-italic).

The isolate nucleic acid underwent whole-genome shotgun sequencing using the Illumina DNA Prep Library Kit (as per manufacturer’s instructions) and was sequenced on an Illumina iSeq100 (2 × 150 bp paired-end). Concurrently, long-read sequencing of the same nucleic acid was performed using an Oxford Nanopore PromethION P2 Solo (R10.4.1 flow cell) and DNA Ligation V14 sequencing kit. Both Illumina and ONT outputs were used for a hybrid genome assembly using long-read scaffolding and short-read polishing, which yielded a single chromosome of 5,983,947 bp (67.6% GC), encoding 5,564 open reading frames, 3 rRNAs, two transfer-messenger RNAs, 15 non-coding RNAs, 55 transfer RNAs, and no CRISPR arrays (Fig. 2B) (12–25). Illumina coverage averaged ~100× with ONT coverage of ~6,300× with estimated genome completeness of 99.2% for bacterial markers (BUSCO) (26) and genus-level genome estimated completeness of 99.94% with 0.72% cross-species contamination (4/693 duplicate gene markers) and 0.00% strain heterogeneity (GTDB-informed marker sets with CheckM) (27).

Analysis of the whole genome identified *Mycobacterium* sp. SMC-2 (NCBI RefSeq: NZ_CP079863.1), a mycobacterial species isolated from sputum from a patient with lung disease in Seoul 2018 (NCBI BioSample: SAMN20167550), with a single complete genome uploaded to NCBI in December 2022 (99.7% average nucleotide identity; Table 1) (28). The most recent common ancestor by taxonomic placement in the reference phylogeny (as well as *de novo* bacterial phylogeny reconstruction) using GTDB r214 and gtdb-tk v.2.3.2 (27, 29), visualized with Interactive Tree of Life (30), was *Mycobacterium nebraskense* (Fig. 2A and C, bold-italic; Table 1). *M. nebraskense* and SMC-2, as well as four other undescribed representative species genomes, form a distinct monophyletic sister clade (Fig. 2A and C, blue) to several described NTM species (yellow). Together with another ancestral clade which included *M. paraffinicum* (green), these genomes form a sister clade to the *M. avium* complex (MAC, purple).

**TABLE 1:** Average nucleotide identity (ANI) with the 10 representative genomes in the GTDB closest to *Mycobacterium* sp*.* unknown

GTDB accession no.	GTDB species	ANI (%)
GCF_002102255.1	*s__Mycobacterium nebraskense*	89.88
GCF_001665605.1	*s__Mycobacterium sp001665605*	89.13
GCF_001667585.1	*s__Mycobacterium sp001667585*	88.87
GCF_001667075.1	*s__Mycobacterium sp001667075*	88.86
GCF_010731595.1	*s__Mycobacterium seoulense*	88.84
GCF_001667735.1	*s__Mycobacterium sp001667735*	88.61
GCF_001667885.1	*s__Mycobacterium scrofulaceum_C*	88.58
GCF_001666835.1	*s__Mycobacterium sp001666835*	88.57
GCF_002102155.1	*s__Mycobacterium europaeum*	88.47
GCF_010731655.1	*s__Mycobacterium paraseoulense*	88.41

Read-level taxonomic profiling with Kraken2 v. 2.1.3/Bracken v. 3.0 (31, 32) and NCBI RefSeq indices showed assignment ambiguity without the *Mycobacterium* sp. SMC-2 genome included, with reads scattered across multiple taxa. At sub-genus level, the largest proportions of reads were assigned to *M. avium* (30.85%) and *Mycobacterium simiae* (12.92%) complexes. At species level, reads were distributed among *M. seoulense* (21.79%), *Mycobacterium paraseoulense* (16.39%), and *M. intracellulare* (10.21%), with remaining reads assigned to various other species. Adding the SMC-2 genome assigned 99.04% of reads to it, confirming identity and highlighting the need for representative reference genomes.

## DISCUSSION

This case highlights the value of WGS in identifying rare and emerging *Mycobacterium* sp., particularly when multi-locus gene testing yields discordant results. Differentiation from *Mycobacterium chimaera* was clinically critical due to its distinct pathogenicity, propensity for disseminated disease, and specific implications for antimicrobial management (33). A known species with multiple database entries was considered more probable than a novel species with a single entry, as the latter could reflect a rare isolate, mislabeling, sequence artifact, contamination, or a true novel species. This frequency-based metric alone was insufficient to resolve the ambiguity, underscoring the necessity of WGS.

Recent genomic advances highlight the insufficiency of 16S rRNA sequencing for species-level identification. Complexes such as MAC and *Mycobacterium abscessus* often share identical 16S sequences despite clinical differences, necessitating multi-locus or WGS to avoid misidentification and to guide appropriate therapy (34–36).

To address non-standardized NTM WGS methods, we confirmed identity using multiple approaches. A closed genome assembly enabled concordant identification using gold-standard GTDB-based phylogenetic analysis. Read-level taxonomic profiling initially scattered across multiple *Mycobacterium* species (*M. avium*, *M. simiae*, *M. seoulense*, *M. paraseoulense*, and *M. intracellulare*) but resolved to 99.04% for SMC-2 after database inclusion. This highlights the necessity of *de novo* genome assembly and inclusion of a representative reference genome, confirming that accurate NTM identification relies on both sequencing depth and reference database comprehensiveness.

This case was prioritized for WGS due to discordance between isolate identification and clinical presentation, with non-response to therapy and a potential healthcare-associated source. Knowledge of the case contributed to the decision to perform WGS; however, this is not always available to laboratories. Despite WGS providing comprehensive identification, cost may limit routine use in public health-funded laboratories. As matrix-assisted laser desorption/ionization time-of-flight databases and NTM resolution improve, this method may offer a fast, low-cost identification alternative for many NTM isolates (37, 38), reserving WGS for complex cases.

This NTM infection was associated with significant morbidity for this child. The burden of invasive surgery, prolonged inpatient admissions, residual pain, and restricted mobility, even after resolution of the infection, has been difficult for the patient and their family.

### Conclusion

We describe a rare NTM causing a significant joint infection in a 5-year-old child, highlighting the role of WGS to resolve diagnostic uncertainties in complex NTM infections. As NTM WGS becomes routine, it is poised to become the gold standard for accurate, timely, and actionable identification in clinical microbiology.

## Data Availability

Sequence data related to this isolate have been deposited into the NCBI GenBank database under BioProject no. PRJNA1340423, BioSample no. SAMN52626330, and accession no. JBSDZO000000000.
